# Prevalence, Serotyping, Molecular Typing, and Antimicrobial Resistance of *Salmonella* Isolated From Conventional and Organic Retail Ground Poultry

**DOI:** 10.3389/fmicb.2018.02653

**Published:** 2018-11-05

**Authors:** Ahmed H. Gad, Usama H. Abo-Shama, Katherine K. Harclerode, Mohamed K. Fakhr

**Affiliations:** ^1^Department of Biological Science, The University of Tulsa, Tulsa, OK, United States; ^2^Microbiology and Immunology Department, Faculty of Veterinary Medicine, Sohag University, Sohag, Egypt

**Keywords:** *Salmonella*, serotyping, antimicrobial resistance, PFGE, plasmids, ground poultry

## Abstract

Ground poultry is marketed as a healthier alternative to ground beef despite the fact that poultry is a major source of foodborne *Salmonella*. The objectives of this study were to determine the prevalence of *Salmonella* in Oklahoma retail ground poultry and to characterize representative isolates by serotyping, antimicrobial resistance, PFGE patterns, and large plasmid profiling. A total of 199 retail ground poultry samples (150 ground turkey and 49 ground chicken) were investigated. The overall prevalence of *Salmonella* in ground poultry was 41% (82/199), and the incidence in conventional samples (47%, 66/141) was higher than in organic samples (27%, 16/58). The prevalence of *Salmonella* in organic ground chicken and organic ground turkey was 33% (3/9) and 26% (13/49), respectively. Twenty six *Salmonella* isolates (19 conventional and 7 organic) were chosen for further characterization. The following six serotypes and number of isolates per serotype were identified as follows: Tennessee, 8; Saintpaul, 4; Senftenberg, 4; Anatum, 4 (one was Anatum_var._15+); Ouakam, 3; and Enteritidis, 3. Resistance to 16 tested antimicrobials was as follows: gentamycin, 100% (26/26); ceftiofur, 100% (26/26); amoxicillin/clavulanic acid, 96% (25/26); streptomycin, 92% (24/26); kanamycin, 88% (23/26); ampicillin, 85% (22/26); cephalothin, 81% (21/26); tetracycline, 35% (9/26); sulfisoxazole, 27% (7/26); nalidixic acid, 15% (4/26); and cefoxitin, 15% (4/26). All isolates were susceptible to amikacin, chloramphenicol, ceftriaxone, and trimethoprim/sulfamethoxazole. All screened isolates were multidrug resistant (MDR) and showed resistance to 4–10 antimicrobials; isolates from organic sources showed resistance to 5–7 antimicrobials. PFGE was successful in clustering the *Salmonella* isolates into distinct clusters that each represented one serotype. PFGE was also used to investigate the presence of large plasmids using S1 nuclease digestion. A total of 8/26 (31%) *Salmonella* isolates contained a ∼100 Kb plasmid that was present in all Anatum and Ouakam isolates. In conclusion, the presence of multidrug resistant *Salmonella* with various serotypes, PFGE profiles, and large plasmids in ground poultry stresses the importance of seeking novel interventions to reduce the risk of this foodborne pathogen. Multidrug resistance (MDR) is considered a high additional risk and continued surveillance at the retail level could minimize the risk for the consumer.

## Introduction

Nontyphoidal *Salmonella* spp. is the primary bacterial pathogen causing foodborne illness and the leading cause of hospitalization among the top five foodborne pathogens in the United States (Scallan et al., 2011). Contaminated meats are the major foodborne sources of *Salmonella*, which has been recovered and characterized from retail beef, pork, bison, chicken, and turkey meats in several countries worldwide (Li et al., 2006; Cetinkaya et al., 2008; Nde et al., 2008; Ammari et al., 2009; Yang et al., 2010; Tafida et al., 2013; Maka et al., 2014; Sallam et al., 2014; Aslam et al., 2012; Soufi et al., 2012; Thai et al., 2012). Consumption of ground poultry has increased in the last few years, partially because it is marketed as a healthier alternative to ground beef. However, ground poultry, particularly ground turkey and chicken, is often contaminated with *Salmonella* (White et al., 2001; Fakhr et al., 2006a; Erol et al., 2013; Cui et al., 2015). A large, multistate-outbreak caused by an antimicrobial-resistant *Salmonella* *enterica* subsp. *enterica* serovar Heidelberg occurred in 2011 from the consumption of contaminated ground turkey and resulted in one death (Folster et al., 2012). Three other multistate-outbreaks caused by *Salmonella* Heidelberg occurred between 2013 and 2014 that were linked to chicken consumption (CDC, 2013a, 2014a,b; Gieraltowski et al., 2016). An outbreak of *Salmonella* *enterica* serovar Stanley infections associated with turkey meat was reported in 10 European countries between 2011 and early 2013 (Kinross et al., 2014). Comparative genomic analysis using Whole Genome Sequencing revealed that the *S.* Heidelberg isolates in the 2011 ground turkey outbreak clustered together when compared to isolates from human, animal, and retail meat sources (Hoffmann et al., 2014). Using an experimental oral challenge experiment in turkey, a recent study showed that the *Salmonella* isolate causing the 2011 outbreak was high in cecal colonization, dissemination to internal organs, and tissue deposition (Nair et al., 2018). Recently, a food-grade essential oil from pimento leaves was shown to reduce attachment of the 2011 *S.* Heidelberg isolate to turkey skin (Nair and Johny, 2017). By testing the host transcriptional response, a recent study showed that young commercial turkeys are susceptible to colonization by *S.* Heidelberg isolated from the 2011 ground turkey outbreak (Bearson et al., 2017).

The presence of antimicrobial resistant *Salmonella* in retail meats, particularly in poultry, is a major risk to the treatment of foodborne illnesses caused by this bacterial pathogen (Antunes et al., 2016; Chai et al., 2017). The presence of multidrug resistant (MDR), nontyphoidal *Salmonella* in retail meats has been reported in several studies (Cetinkaya et al., 2008; Zhao et al., 2008; Yildirim et al., 2011; Thai et al., 2012; Van et al., 2012; Maka et al., 2014; Yang et al., 2014; Iwamoto et al., 2017; Clothier et al., 2018). Most of the antimicrobial resistance genes in *Salmonella* are carried on conjugative plasmids that facilitate transfer between different isolates (Jones and Stanley, 1992; Rotgers and Casadesüs, 1999; Carattoli, 2003; Rychlik et al., 2006). Conjugation experiments showed that 95% of the β–lactamase genes (bla_CMY_) in *Salmonella* are plasmid-encoded (Folster et al., 2011). Quinolone resistance genes were also plasmid-borne in *Salmonella* isolated from human cases in the United States (Sjölund-Karlsson et al., 2010). Three emerging European clones of *Salmonella enterica* subsp. *enterica* serovar Typhimurium circulating in Europe were found to harbor MDR plasmids that encode additional virulence functions (García et al., 2014). Plasmid profiling is often used in epidemiological studies related to surveillance of disease outbreaks and in tracing the dissemination of antibiotic resistance (Mayer, 1988).

Pulsed field gel electrophoresis (PFGE) is considered the gold standard in typing *Salmonella* and is known for its ability to discriminate isolates and for tracking the source of outbreaks (Tenover et al., 1995; Fakhr et al., 2005; Foley et al., 2006, 2009; Folster et al., 2012). PFGE profiling has been used with relative success as a method to identify *Salmonella* serotypes (Gaul et al., 2007; Zou et al., 2010). A meta-analysis of PFGE fingerprints based on a constructed *Salmonella* database of 45,923 PFGE patterns indicated the presence of serotype-specific patterns that may potentially reduce the need to perform the laborious, traditional serotyping (Zou et al., 2013).

Despite the risk associated with the consumption of ground poultry contaminated with *Salmonella*, studies investigating the prevalence and characterization of *Salmonella* in retail ground poultry are relatively scarce. The objectives of this study were to determine the prevalence of *Salmonella* in retail ground poultry sold in the Tulsa, Oklahoma area and to characterize a selected number of the recovered strains by serotyping, antimicrobial resistance screening, plasmid profiling, and PFGE.

## Materials and Methods

### Bacterial Sampling and Identification

Conventional methods were used to isolate *Salmonella* from ground turkey as described previously (Fakhr et al., 2006a; Nde et al., 2008). In the summer of 2009, 199 samples of ground poultry meat (150 and 49 from turkey and chicken, respectively) representing five brands were purchased at six retail stores representing six supermarket chains in Tulsa, Oklahoma. Ground poultry samples were stored in chilled containers, and transported to the laboratory within 4 h. Each sample (25 g) was subjected to a pre-enrichment step by combining it with 225 mL of Buffered Peptone Water (BPW) (EMD, Gibbstown, NJ, United States) in sterile plastic bags (VWR Scientific, Radnor, PA, United States); the samples was massaged briefly by hand for 5 min. The pre-enrichment rinsate was then incubated at 37°C for 24 h. To selectively enrich for *Salmonella*, 0.1 and 0.5 mL of each pre-enrichment broth sample was transferred to 10 mL of Rappaport-Vassiliadis broth (RVB; Difco, Becton Dickinson, Sparks, MD, United States) and tetrathionate broth (TTB; Difco, Becton Dickinson, Sparks, MD, United States), respectively, and incubated at 42°C for 24 h. The pre-enrichment broths of duplicate samples were then artificially spiked with 10 μL of an overnight broth of two *Salmonella* strains (one H_2_S-positive and one H_2_S-negative); these served as positive controls. After selective enrichment was completed, a loopful of broth contains each of the enriched samples, including the two artificially-spiked *Salmonella* positive controls, were inoculated by dilution-streaking onto two selective agar media, XLT4 (Difco, Becton Dickinson, Sparks, MD, United States) and Brilliant Green Sulfide (BGS) (Difco, Becton Dickinson, Sparks, MD, United States) and incubated at 37°C for 24 h. The identity of 4–6 suspected *Salmonella* colonies from each sample were confirmed biochemically by dilution streaking onto Triple Sugar Iron Agar (TSI) (Difco, Becton Dickinson, Sparks, MD, United States) and Lysine Iron Agar slants (Difco, Becton Dickinson, Sparks, MD, United States) and incubated at 37°C for 24 h. Suspected *Salmonella* isolates were subjected to molecular confirmation by PCR using *invA* as described below.

The *invA* gene was amplified using the following PCR primers: forward, 5′**-** GTGAAATTATCGCCACGTTCGGGCAA-3′; and reverse, 5′**-** TCATCGCACCGTCAAAGGAACC-3′ as described previously (Rahn et al., 1992). PCR was conducted in 25 μL reaction volumes containing the following: 12.5 μL GoTaq^®^ Green Master Mix (Promega, Madison, WI, United States), 3.5 μL sterile water (Promega, Madison, WI, United States), 1 μL (25 pmol) of each primer (IDT, Coralville, IA, United States), and 3 μL of template DNA. The cycling conditions were as follows: (1) 95°C for 5 min; (2) 94°C for 1 min; (3) 55°C for 1 min; (4) 72°C for 1 min; and (5) 72°C for 10 min. Steps 2 through 4 were repeated for 35 cycles. PCR products were subjected to agarose gel electrophoresis, and a 1 kb plus DNA ladder (Bioneer, Alameda, CA, United States) was used as a molecular marker. Gel images were taken using a Bio-Rad Gel DocTM XR UV gel documentation system (Bio-Rad, Hercules, CA, United States). The presence of the 284 bp *invA* PCR product was considered to be positive for *Salmonella* molecular identification. Once confirmed as *Salmonella*, one isolate was kept as a representative for each ground poultry sample and further characterized by serotyping, antibiotic resistance profile, PFGE, and plasmid content.

### Serotyping

*Salmonella* isolates selected for serotyping were given a serial designation from GP001 to GP023 and from GP025 to GP027. Isolates were sent to the National Veterinary Service Laboratory (NVSL) in Ames, Iowa, for serotyping.

### Antibiotic Resistance Screening

*Salmonella* isolates were subjected to antimicrobial resistance profiling using the following 16 antimicrobials: cefoxitin (FOX), amikacin (AMI), chloramphenicol (CHL), tetracycline (TET), ceftriaxone (CTR), amoxicillin/clavulanic acid (AMC), ciprofloxacin (CIP), gentamycin (GEN), nalidixic acid (NAL), ceftiofur (TIO), sulfisoxazole (FIS), trimethoprim/sulfamethoxazole (SXT), cephalothin (CEP), kanamycin (KAN), ampicillin (AMP), and streptomycin (STR). Isolates were grown on Mueller-Hinton (MH) agar (Difco) and incubated for 24 h at 37°C. Cultures were then added to Mueller-Hinton broth (Difco), and the turbidity was adjusted to a 0.5 McFarland standard, and inoculated onto 6-inch MH agar plates supplemented with the appropriate antimicrobials. Multiple antibiotic concentrations were tested including the breakpoint established for each antimicrobial according to the Clinical and Laboratory Standards Institute (CLSI) (Cockerill, 2011). The ranges of the concentrations used and the breakpoint of each of the 16 antimicrobials tested in this study were detailed previously (Fakhr et al., 2006b). Plates were then incubated at 37°C for 48 h; results were read for growth or no growth and denoted as resistant or susceptible, respectively, according to the breakpoints for each antimicrobial.

### PFGE

Plug preparation for PFGE profiling was performed according to the PulseNet protocol and conditions established by the CDC (CDC, 2013b). Slices of the prepared PFGE plugs (2-mm wide) were incubated with *Xba*I (Promega, Madison, WI, United States) at a concentration of 50 U/plug for 3 h at 37°C. Plug slices were then inserted into the wells of 1% Seakem Gold Agarose gels. *Xba*I-digested *Salmonella* serovar Braenderup H9812 was used as a sizing marker. PFGE was conducted in a CHEF Mapper PFGE system (Bio-Rad, Hercules, CA, United States) for 18 h following the electrophoresis conditions established for *Salmonella* by the PulseNet protocol; these included an initial switch time of 2.16 s, and a final switch time of 63.8 s (CDC, 2013b). After electrophoresis, gel images were captured using a Bio-Rad Gel DocTM XR UV gel documentation system (Bio-Rad, Hercules, CA, United States). Images were then imported and analyzed using the BioNumerics software v. 6.6 (Applied Maths, Austin, TX, United States). Similarity analysis and the banding patterns were analyzed using the Dice coefficient and clustered using the unweighted pair group method with arithmetic mean (UPGMA) and a 1.5% band tolerance.

### Plasmid Detection

Screening of large plasmids was performed by PFGE as described previously (Barton et al., 1995; Marasini and Fakhr, 2014). The PFGE plugs were prepared as described above; thin slices were cut and digested with S1 nuclease (17 IU/plug) for 45 min at 37°C to linearize the plasmids. Plug slices were then inserted into the wells of 1% Seakem Gold Agarose gels, and *Xba*I-digested *Salmonella* serovar Braenderup H9812 was used as a sizing marker. PFGE was conducted using the CHEF Mapper PFGE system for 16 h using the conditions established for *Salmonella* by the PulseNet protocol (CDC, 2013b).

Large plasmids detected by PFGE were isolated by alkaline lysis using the Qiagen Miniprep kit and protocols established for Gram-negative bacteria (Qiagen Inc., Valencia, CA, United States). Isolated plasmids were analyzed by electrophoresis in 0.8% agarose gels at 120 V for 2 h. Gels were stained with ethidium bromide for 45 min, and images were captured using the Bio-Rad gel documentation system. DNA markers for sizing included plasmids preps of *E. coli* strains NCTC 50192 and NCTC 50193 and the 1 Kb plus DNA ladder (Bioneer). The isolated plasmids were also digested with *Eco*RI and *Hin*dIII (Promega, Madison, WI, United States) and subjected to agarose gel electrophoresis to determine variable restriction patterns.

## Results

### Prevalence of *Salmonella* in Ground Poultry

A total of 199 retail ground poultry samples were investigated in this study (Table 1). Although only 6% (9/150) of the ground turkey samples were organic, all 49 ground chicken samples were organic. The overall prevalence of *Salmonella* in ground poultry was 41% (82/199), whereas the prevalence in conventional samples (47%; 66/141) was higher than organic samples (27%; 16/58) (Table 1). The prevalence of *Salmonella* in organic ground chicken was 26% (13/49), whereas the incidence in ground turkey was 47% (66/141) and 33% (3/9) for conventional and organic samples, respectively (Table 1).

**Table 1:** Prevalence of *Salmonella* in ground poultry samples collected in this study.

Prevalence of *Salmonella* in ground poultry									

	Ground turkey			Ground chicken			Total ground poultry		
			
	Conventional ^∗^np/n (%)	Organic np/n (%)	Total np/n (%)	Conventional np/n (%)	Organic np/n (%)	Total np/n (%)	Conventional np/n (%)	Organic np/n (%)	Total np/n (%)
*Salmonella*	66/141 (47)	3/9 (33)	69/150 (46)	0/0 (0)	13/49 (26)	13/49 (26)	66/141 (47)	16/58 (27)	82/199 (41)


*^∗^np, number of positive samples; n, number of samples collected.*

### Serotyping

To reduce the cost, twenty six isolates representing unique *Salmonella-*positive ground poultry samples (19 conventional, 7 organic) were selected for further characterization by serotyping, antibiotic resistance profiling, PFGE, and plasmid profiling. The twenty six isolates were carefully chosen to fairly represent the eighty two positive samples in this study in regards to variation in the collection and expiration date, brand, supermarket chain and location, and meat source (ground turkey or ground chicken). Six serotypes were identified: Tennessee (8 isolates), Saintpaul (4 isolates), Senftenberg (4 isolates), Anatum (4 isolates, including one Anatum_var._15+), Ouakam (3 isolates), and Enteritidis (3 isolates) (Table 2). Serotypes Saintpaul, Ouakam, and Anatum were detected in conventional ground turkey, but not in organic ground chicken; the latter contained serotypes Tennessee (*n* = 4), Enteritidis (*n* = 2), and Senftenberg (*n* = 1) (Table 2).

**Table 2:** Ground poultry sources, serotypes and large plasmid profiles of the 26 *Salmonella* isolates characterized in this study.

Isolate #	Turkey/Chicken	Conventional/Organic	Serotype	Large plasmids
GP001	Ground turkey	Conventional	Anatum_var._15 +	∼100 kb^∗^
GP002	Ground turkey	Conventional	Anatum	∼100 kb^∗^
GP003	Ground turkey	Conventional	Saintpaul	∼100 kb
GP004	Ground turkey	Conventional	Saintpaul	
GP005	Ground chicken	Organic	Tennessee	
GP006	Ground chicken	Organic	Tennessee	
GP007	Ground turkey	Conventional	Saintpaul	
GP008	Ground turkey	Conventional	Senftenberg	
GP009	Ground turkey	Conventional	Ouakam	∼100 kb^∗∗^
GP010	Ground turkey	Conventional	Saintpaul	
GP011	Seasoned ground turkey	Conventional	Tennessee	
GP012	Ground turkey	Conventional	Anatum	∼100 kb^∗^
GP013	Turkey breakfast sausage	Conventional	Ouakam	∼100 kb^∗∗^
GP014	Ground turkey	Conventional	Tennessee	
GP015	Ground turkey	Conventional	Tennessee	
GP016	Ground turkey	Conventional	Ouakam	∼100 kb^∗∗^
GP017	Ground chicken	Organic	Senftenberg	
GP018	Ground chicken	Organic	Tennessee	
GP019	Ground chicken	Organic	Tennessee	
GP020	Ground turkey breast	Conventional	Tennessee	
GP021	Ground chicken	Organic	Enteritidis	
GP022	Ground chicken	Organic	Enteritidis	
GP023	Ground turkey	Conventional	Anatum	∼100 kb^∗^
GP025	Ground turkey	Conventional	Enteritidis	
GP026	Ground turkey	Conventional	Senftenberg	
GP027	Ground turkey	Conventional	Senftenberg	


*^∗^Large plasmids in strains GP001, GP002, GP012, and GP023 share the same restriction pattern. ^∗∗^Large plasmids in strains GP009, GP013, and GP016 share the same restriction pattern.*

### Antibiotic Resistance

**FIGURE 1:**
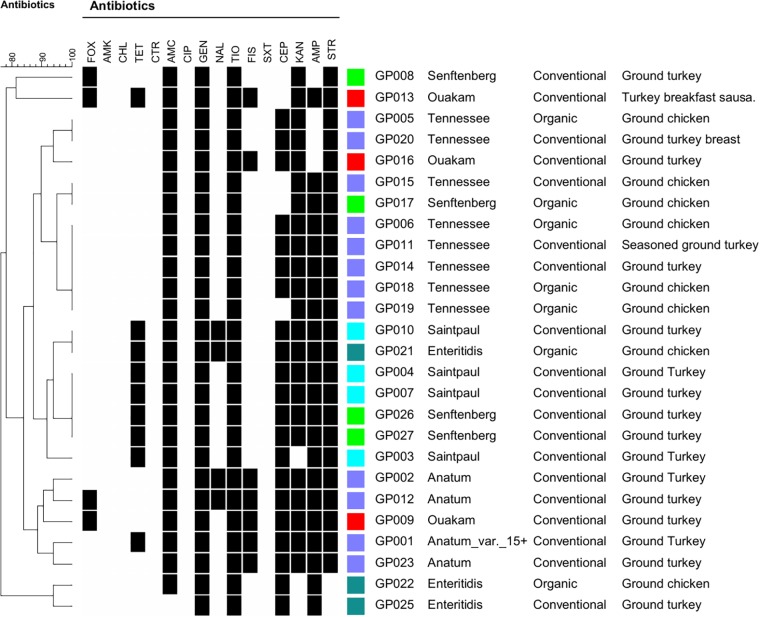
Dendrogram of 26 *Salmonella* strains showing antibiotic resistance profiles, serotypes, and sources of ground poultry. Clustering was based on antibiotic resistance profiling, and the dendogram was created using BioNumerics software. Black squares indicate resistance. Abbreviations: FOX, cefoxitin; AMK, amikacin; CHL, chloramphenicol: TET, tetracycline; CTR, ceftiaxone; AMC, amoxicillin-clavulanic acid; CIP, ciprofloxac; GEN, gentamicin; NAL, nalidixic acid; TIO, ceftiofur; FIS, sulfisoxazole; SXT, trimethoprim/sulfamethoxazole; CEP, cephalothin, KAN, kanamycin; AMP, ampicillin; STR, streptomycin.

The 26 serotyped *Salmonella* isolates were subjected to antibiotic resistance profiling to 16 antimicrobials (Figure 1). All 26 isolates were resistant to both gentamycin and ceftiofur. Resistance to the remaining antimicrobials was as follows: amoxicillin/clavulanic acid, 96% (25/26); streptomycin, 92% (24/26); kanamycin, 88% (23/26); ampicillin, 85% (22/26); cephalothin, 81% (21/26); tetracycline, 35% (9/26 ), sulfisoxazole 27% (7/26); nalidixic acid 15% (4/26), and cefoxitin, 15% (4/26). All isolates were susceptible to amikacin, chloramphenicol, ceftriaxone, and trimethoprim/sulfamethoxazole. All 26 tested isolates were multidrug-resistant (MDR) and exhibited resistance to 4–10 antimicrobials (Figure 1). Isolates from organic sources also exhibited MDR to 5–7 antimicrobials. Sulfisoxazole resistance was observed only in the Anatum and Ouakam serotypes. Although there was some variability, some antibiotic profiles were common among a particular serotype (Figure 1).

### PFGE Analysis

**FIGURE 2:**
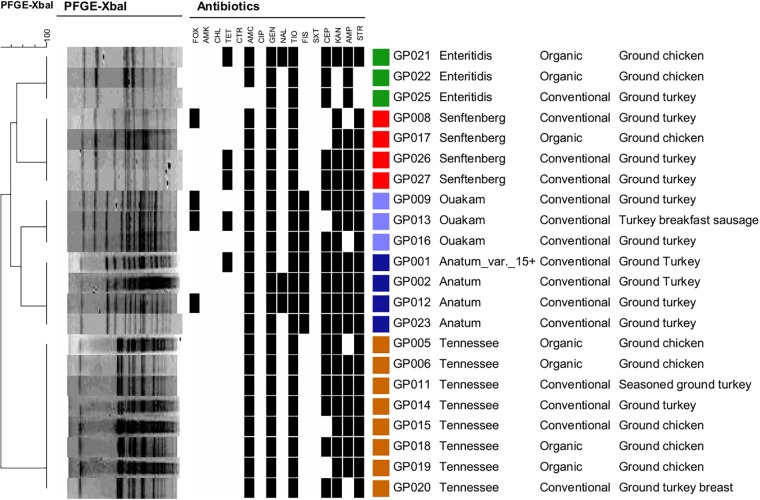
Dendogram and pulsed field gel electrophoresis (PFGE) profiling of 22 *Salmonella* isolates using *Xba*I. Antibiotic resistance, serotypes, and the ground poultry sources are shown for the 22 PFGE-typable *Salmonella* strains. Similarity analysis was performed using the Dice coefficient, and clustering was performed using UPGMA based on PFGE profiles. Black squares indicate resistance. FOX, cefoxitin; AMK, amikacin; CHL, chloramphenicol: TET, tetracycline; CTR, ceftiaxone; AMC, amoxicillin-clavulanic acid; CIP, ciprofloxac; GEN, gentamicin; NAL, nalidixic acid; TIO, ceftiofur; FIS, sulfisoxazole; SXT, trimethoprim/sulfamethoxazole; CEP, cephalothin, KAN, kanamycin; AMP, ampicillin; STR, streptomycin.

All 26 serotyped *Salmonella* isolates were subjected to PFGE to determine *Xba*I restriction patterns. Although the four Saintpaul isolates were non-typable by *Xba*I-mediated PFGE, the remaining 22 isolates representing the other five serotypes were successfully analyzed (Figure 2). PFGE grouped the 22 *Salmonella* isolates into five distinct clusters each representing one of the following five serotypes: Enteritidis, Senftenberg, Ouakam, Anatum (including the Anatum_var._15+), and Tennessee.

### Plasmid Profiling

**FIGURE 3:**
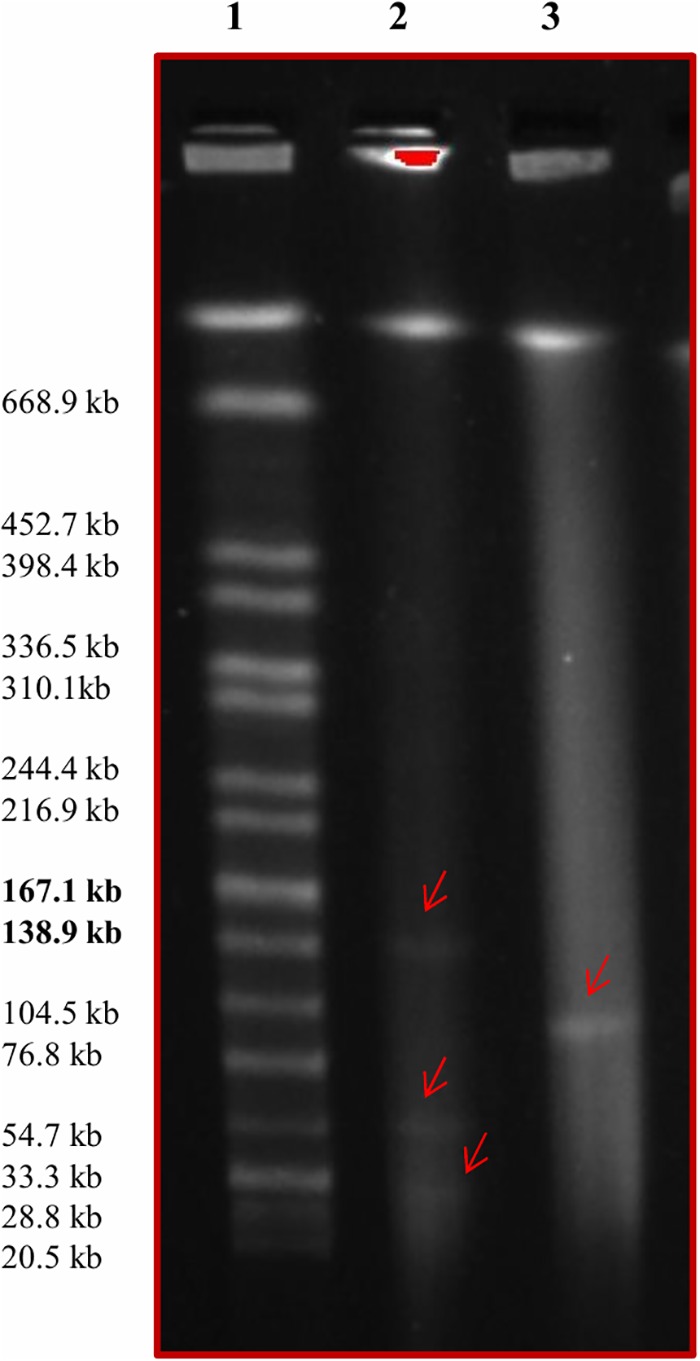
Pulsed field gel electrophoresis image showing the presence of large plasmids. Lane 1 contains the *Salmonella* serovar Braenderup H9812 molecular marker. Lane 2 contains *E. coli* NCTC 50192 treated with S1 nuclease; the three large plasmids (147, 63, and 43.5 kb) are indicated with red arrows. Lane 3 contains *Salmonella* Ouakam strain GP016 treated with S1 nuclease; the red arrow indicates the presence of a ∼100 kb plasmid.

PFGE was used to investigate the presence of large plasmids in the 26 serotyped isolates using S1 nuclease digestion. Eight of the 26 *Salmonella* isolates contained a large ∼100 Kb plasmid (Table 2). The four serotype Anatum strains, including the Anatum_var._15 + isolate, contained a ∼100 Kb plasmid, as did the three Ouakam isolates and one of the four Saintpaul isolates (Table 2). A PFGE gel showing the 100 Kb plasmid in one of the *Salmonella* Ouakam isolates is presented in Figure 3 (lane 3). All eight isolates harboring large plasmids were isolated from conventional samples. Restriction digestion analysis using *Eco*RI and/or *Hin*dIII revealed that the large plasmids harbored by the four Anatum isolates had similar restriction patterns (Table 2). Likewise, similar restriction patterns were obtained for large plasmids of the three Ouakam isolates (Table 2).

## Discussion

*Salmonella* was recovered from 41% (82/199) of ground poultry samples collected in this study, which stresses the importance of monitoring this foodborne pathogen at the retail level. This result is similar to another study where *Salmonella* was recovered from 40% (30/74) of ground turkey samples in Fargo, North Dakota (Fakhr et al., 2006b). In a larger study conducted by researchers at the FDA, *Salmonella* prevalence was 52% in ground turkey after screening 1,499 ground turkey samples collected from grocery stores in several FoodNet sites across the United States (Zhao et al., 2006). A recent study indicated that the high incidence of *Salmonella* in turkey neck skin may predict a flock with greater potential for *Salmonella* contamination of ground turkey (Cui et al., 2015). Another study found that preharvest screening of *Salmonella* using a rapid protocol could potentially reduce *Salmonella* in ground turkey meat and possibly decrease future salmonellosis outbreaks (Evans et al., 2015). Recently, the *Salmonella* lytic bacteriophage preparation (SalmoFresh) was investigated for efficacy in reducing *Salmonella* populations on turkey breast cutlets and ground turkey (Sharma et al., 2015). While the bacteriophage preparation was effective in reducing *Salmonella* on turkey breast cutlets, it did not reduce the incidence of *Salmonella* Heidelberg in ground turkey (Sharma et al., 2015).

The detection of six serotypes in the 26 *Salmonella*-positive isolates indicates a high level of *Salmonella* diversity in ground turkey. This variability was also observed in other studies where different serotypes were detected depending on the geographic location and the date when studies were conducted (Fakhr et al., 2006b; Erol et al., 2013). All isolates were susceptible to amikacin, chloramphenicol, trimethoprim/sulfamethoxazole, and ceftriaxone; the latter is particularly important because ceftriaxone is the drug of choice for treating salmonellosis in children (White et al., 2001; Iwamoto et al., 2017). A recent study examining NARMS data between 1996 and 2013 showed that ceftriaxone resistance in *Salmonella* isolated from humans correlated with resistance in retail meats and food animals in the United States (Iwamoto et al., 2017). In the present study, the high percentages of resistance for gentamycin (100%), ceftiofur (100%), amoxicillin/clavulanic acid (96%), streptomycin (92%), kanamycin (88%), ampicillin (85%), and cephalothin (81%) is alarming. Similar high percentages of resistance ranging from 91.6 to 100% to several of these antimicrobials were reported in *Salmonella* isolated from chicken meat and giblets collected from Mansoura, Egypt (Abd-Elghany et al., 2015). The high incidence of resistance to aminoglycoside and β-lactam antibiotics is coincident with high prevalence of *S. enterica* resistance to these two classes of antibiotics in food animals (Foley and Lynne, 2008; Frye and Jackson, 2013). Ceftiofur has been used to prevent the death of 1-day old turkey poults, and its use in animal feed might select for the acquisition of plasmids with antibiotic resistance (Wittum, 2012). In our study, the moderate level of resistance in screened *Salmonella* isolates to tetracycline (35%), sulfisoxazole (27%), nalidixic acid (15%), and cefoxitin (15%) has been documented in other studies (Aslam et al., 2012; Thai et al., 2012; Nisar et al., 2017). A recent study analyzed the surveillance data of 18 years on antimicrobial resistance profiling showed higher level of resistance of chicken breast isolates toward third-generation cephalosporins and tetracyclines when compared to human isolates (Paudyal et al., 2018). It is noteworthy that all 26 isolates in this study, including those isolated from organic sources, exhibited multidrug resistance (MDR). The presence of MDR *Salmonella* in retail meats in the United States, Canada, and the European Union is well- established (White et al., 2001; Zhao et al., 2006; Aslam et al., 2012; Florez-Cuadrado et al., 2018). In a recent study, 36% of *Salmonella* isolates were multidrug resistant to two to five antimicrobials despite being isolated from a antimicrobials free turkey production facility (Sanad et al., 2016). In a large study conducted in Spain, 41% of *Salmonella* isolates from meat products was resistant to three or more antibiotics (Doménech et al., 2015). The high number of MDR *Salmonella* detected in retail meats sold in Oklahoma is not surprising since previous studies have shown the high incidence of MDR *Campylobacter* spp. and *Staphylococcus aureus* in Oklahoma retail meats (Noormohamed and Fakhr, 2012, 2013, 2014; Abdalrahman and Fakhr, 2015; Abdalrahman et al., 2015a,b).

In this study, PFGE successfully grouped *Salmonella* isolates into distinct clusters that represented individual serotypes. PFGE previously showed discriminative ability for some *Salmonella* serotypes and antimicrobial resistance profiles (Fakhr et al., 2006b; Zhao et al., 2006). PFGE profiling was considered a possible alternative for identification of some *Salmonella* serotypes (Gaul et al., 2007; Zou et al., 2010, 2013). In a recent study, PFGE showed that *Salmonella* Heidelberg isolates from turkeys were more genetically diverse than those isolated from chickens (Nisar et al., 2017). In this study, PFGE in combination with S1 nuclease digestion enabled successful detection of large plasmids in 8/26 (31%) of the *Salmonella* isolates. A large plasmid of ∼100 Kb was detected in all Anatum and Ouakam isolates and one Saintpaul isolate; furthermore, the Anatum and Ouakam were the only isolates with sulfisoxazole resistance, which might indicate a role for these plasmids in mediating resistance to this antimicrobial. Recently, we released the whole genome sequences of three isolates described in this study including *Salmonella* Ouakam, Anatum, and Anatum var. 15+; these isolates harbored large plasmids of 109,715, 112,176, and 112,176 bp, respectively (Marasini et al., 2016a,b). The large plasmids in the Anatum and Anatum_var._15+ isolates were identical in size, which is consistent with the restriction patterns observed in this study. The large plasmids in *Salmonella* are known to encode genes for virulence and MDR, and their conjugative properties facilitates dissemination of virulence and antimicrobial resistance (Carattoli, 2003; Rychlik et al., 2006; Sajid and Schwarz, 2009; Folster et al., 2011; García et al., 2014).

## Conclusion

In conclusion, the presence of MDR *Salmonella* with various serotypes, PFGE profiles, and large plasmids in ground poultry is alarming. Intervention strategies to reduce this important foodborne pathogen in retail meats are imperative, particularly in ground turkey. While ground poultry is being marketed as a healthier alternative to ground beef, consumers should apply strict food safety practices when handling ground turkey and consider cooking the meat thoroughly. The high prevalence of *Salmonella* strains recovered in this study with resistance to several antimicrobials can complicate the treatment of salmonellosis and increase the risk of this human illness. This is particularly critical for treating children with salmonellosis, since ceftriaxone is the drug of choice for pediatric salmonellosis and resistance to this compound would derail the efficacy of this antibiotic.

## Author Contributions

MF performed the research design and provided the laboratory supplies. AG, U-AS, KH, and MF carried out the bench work and data analysis. MF and AG prepared the manuscript.

## Conflict of Interest Statement

The authors declare that the research was conducted in the absence of any commercial or financial relationships that could be construed as a potential conflict of interest.

## References

[B1] Abdalrahman L. Fakhr M. (2015). Incidence, antimicrobial susceptibility, and toxin genes possession screening of *Staphylococcus aureus* in retail chicken livers and gizzards. *Foods* 4 115–129. 10.3390/foods4020115 28231192 PMC5302321

[B2] Abdalrahman L. S. Stanley A. Wells H. Fakhr M. K. (2015a). Isolation, virulence, and antimicrobial resistance of methicillin-resistant *Staphylococcus aureus* (MRSA) and methicillin sensitive *Staphylococcus aureus* (MSSA) strains from Oklahoma retail poultry meats. *Int. J. Environ. Res. Public Health* 12 6148–6161. 10.3390/ijerph120606148 26035662 PMC4483693

[B3] Abdalrahman L. S. Wells H. Fakhr M. (2015b). *Staphylococcus aureus* is more prevalent in retail beef livers than in pork and other beef cuts. *Pathogens* 4 182–198. 10.3390/pathogens4020182 25927961 PMC4493469

[B4] Abd-Elghany S. M. Sallam K. I. Abd-Elkhalek A. Tamura T. (2015). Occurrence, genetic characterization and antimicrobial resistance of *Salmonella* isolated from chicken meat and giblets. *Epidemiol. Infect.* 143 997–1003. 10.1017/S0950268814001708 25004116 PMC9507119

[B5] Ammari S. Laglaoui A. En-Nanei L. Bertrand S. Wildemauwe C. Barrijal S. (2009). Isolation, drug resistance and molecular characterization of *Salmonella* isolates in northern Morocco. *J. Infect. Dev. Ctries.* 3 41–49. 10.3855/jidc.104 19749448

[B6] Antunes P. Mourão J. Campos J. Peixe L. (2016). Salmonellosis: the role of poultry meat. *Clin. Microbiol. Infect.* 22 110–121. 10.1016/j.cmi.2015.12.004 26708671

[B7] Aslam M. Checkley S. Avery B. Chalmers G. Bohaychuk V. Gensler G. (2012). Phenotypic and genetic characterization of antimicrobial resistance in *Salmonella* serovars isolated from retail meats in Alberta, Canada. *Food Microbiol.* 32 110–117. 10.1016/j.fm.2012.04.017 22850381

[B8] Barton B. M. Harding G. P. Zuccarelli A. J. (1995). A general method for detecting and sizing large plasmids. *Anal. Biochem.* 226 235–240. 10.1006/abio.1995.1220 7793624

[B9] Bearson B. L. Bearson S. M. D. Looft T. Cai G. Shippy D. C. (2017). Characterization of a multidrug-resistant *Salmonella enterica* serovar Heidelberg outbreak strain in commercial turkeys: colonization, transmission, and host transcriptional response. *Front. Vet. Sci.* 4:156. 10.3389/fvets.2017.00156 28993809 PMC5622158

[B10] Carattoli A. (2003). Plasmid-mediated antimicrobial resistance in *Salmonella enterica*. *Curr. Issues Mol. Biol.* 5 113–122. 12921226

[B11] CDC (2013a). *CDC - Salmonella Heidelberg Infections Linked to Chicken - Salmonella - Feb, 2013*. Available at: https://www.cdc.gov/salmonella/heidelberg-02-13/index.html [accessed September 19, 2018].

[B12] CDC (2013b). Standard operating procedure for PulseNet PFGE of *Escherichia coli* O157: H7, *Escherichia coli* non - O157 (STEC), *Salmonella* serotypes, *Shigella sonnei* and *Shigella flexneri*. *E. coli Salmonella* Detect. *Meat* 157 1–13. 10.1089/fpd.2006.3.59 16602980

[B13] CDC (2014a). *Multistate Outbreak of Multidrug-Resistant Salmonella Heidelberg Infections Linked to Foster Farms Brand Chicken (Final Update)*, 1–18. Available at: https://www.cdc.gov/salmonella/heidelberg-10-13/index.html [accessed September 19, 2018].

[B14] CDC (2014b). *Salmonella Heidelberg Infections Linked to Tyson Brand Mechanically Separated Chicken at a Correctional Facility | February 2014 | Salmonella | CDC*. Available at: https://www.cdc.gov/salmonella/heidelberg-01-14/index.html [accessed September 19, 2018].

[B15] Cetinkaya F. Cibik R. Ece Soyutemiz G. Ozakin C. Kayali R. Levent B. (2008). *Shigella* and *Salmonella* contamination in various foodstuffs in Turkey. *Food Control* 19 1059–1063. 10.1016/j.foodcont.2007.11.004

[B16] Chai S. J. Cole D. Nisler A. Mahon B. E. (2017). Poultry: the most common food in outbreaks with known pathogens, United States, 1998-2012. *Epidemiol. Infect.* 145 316–325. 10.1017/S0950268816002375 27780481 PMC9507623

[B17] Clothier K. A. Kim P. Mete A. Hill A. E. (2018). Frequency, serotype distribution, and antimicrobial susceptibility patterns of *Salmonella* in small poultry flocks in California. *J. Vet. Diagn. Investig.* 30 471–475. 10.1177/1040638718755418 29405899 PMC6505821

[B18] Cockerill F. (2011). *Performance Standards for Antimicrobial Susceptibility Testing: Twenty-First Informational Supplement*, Vol. 31 Wayne, PA: CLSI, 1–172

[B19] Cui Y. Guran H. S. Harrison M. A. Hofacre C. L. Alali W. Q. (2015). *Salmonella* levels in turkey neck skins, drumstick bones, and spleens in relation to ground turkey. *J. Food Prot.* 78 1945–1953. 10.4315/0362-028X.JFP-15-240 26555516

[B20] Doménech E. Jiménez-Belenguer A. Pérez R. Ferrús M. A. Escriche I. (2015). Risk characterization of antimicrobial resistance of *Salmonella* in meat products. *Food Control* 57 18–23. 10.1016/j.foodcont.2015.04.001

[B21] Erol I. Goncuoglu M. Ayaz N. D. Ellerbroek L. Bilir Ormanci F. S. Iseri Kangal O. (2013). Serotype distribution of *Salmonella* isolates from turkey ground meat and meat parts. *Biomed Res. Int.* 2013:281591. 10.1155/2013/281591 23936785 PMC3722973

[B22] Evans N. P. Evans R. D. Regalado J. Sullivan J. F. Dutta V. Elvinger F. (2015). Preharvest *Salmonella* detection for evaluation of fresh ground poultry product contamination. *J. Food Prot.* 78 1266–1271. 10.4315/0362-028X.JFP-14-509 26197276

[B23] Fakhr M. K. McEvoy J. M. Sherwood J. S. Logue C. M. (2006a). Adding a selective enrichment step to the iQ-Check^TM^ real-time PCR improves the detection of *Salmonella* in naturally contaminated retail turkey meat products. *Lett. Appl. Microbiol.* 43 78–83. 10.1111/j.1472-765X.2006.01903.x 16834725

[B24] Fakhr M. K. Sherwood J. S. Thorsness J. Logue C. M. (2006b). Molecular characterization and antibiotic resistance profiling of *Salmonella* isolated from retail Turkey meat products. *Foodborne Pathog. Dis.* 3 366–374. 10.1089/fpd.2006.3.366 17199518

[B25] Fakhr M. K. Nolan L. K. Logue C. M. (2005). Multilocus sequence typing lacks the discriminatory ability of pulsed-field gel electrophoresis for typing *Salmonella enterica* serovar typhimurium. *J. Clin. Microbiol.* 43 2215–2219. 10.1128/JCM.43.5.2215-2219.2005 15872244 PMC1153745

[B26] Florez-Cuadrado D. Moreno M. A. Ugarte-Ruíz M. Domínguez L. (2018). Antimicrobial resistance in the food chain in the European union. *Adv. Food Nutr. Res.* 86 115–136. 10.1016/bs.afnr.2018.04.004 30077219

[B27] Foley S. L. Lynne A. M. (2008). Food animal-associated *Salmonella* challenges: pathogenicity and antimicrobial resistance. *J. Anim. Sci.* 86(Suppl. 14), E173–E187. 10.2527/jas.2007-0447 17878285

[B28] Foley S. L. Lynne A. M. Nayak R. (2009). Molecular typing methodologies for microbial source tracking and epidemiological investigations of Gram-negative bacterial foodborne pathogens. *Infect. Genet. Evol.* 9 430–440. 10.1016/j.meegid.2009.03.004 19460308

[B29] Foley S. L. White D. G. McDermott P. F. Walker R. D. Rhodes B. Fedorka-Cray P. J. (2006). Comparison of subtyping methods for differentiating *Salmonella enterica* serovar typhimurium isolates obtained from food animal sources. *J. Clin. Microbiol.* 44 3569–3577. 10.1128/JCM.00745-06 17021084 PMC1594788

[B30] Folster J. P. Pecic G. McCullough A. Rickert R. Whichard J. M. (2011). Characterization of *bla* CMY -encoding plasmids among *Salmonella* isolated in the United States in 2007. *Foodborne Pathog. Dis.* 8 1289–1294. 10.1089/fpd.2011.0944 21883005

[B31] Folster J. P. Pecic G. Rickert R. Taylor J. Zhao S. Fedorka-Cray P. J. (2012). Characterization of multidrug-resistant *Salmonella enterica* serovar Heidelberg from a ground Turkey-associated outbreak in the United States in 2011. *Antimicrob. Agents Chemother.* 56 3465–3466. 10.1128/AAC.00201-12 22450975 PMC3370747

[B32] Frye J. G. Jackson C. R. (2013). Genetic mechanisms of antimicrobial resistance identified in *Salmonella enterica, Escherichia coli*, and *Enteroccocus* spp. isolated from U.S. food animals. *Front. Microbiol.* 4:135. 10.3389/fmicb.2013.00135 23734150 PMC3661942

[B33] García P. Hopkins K. L. García V. Beutlich J. Mendoza M. C. Threlfall J. (2014). Diversity of plasmids encoding virulence and resistance functions in *Salmonella enterica* subsp. *enterica* serovar typhimurium monophasic variant 4,[5],12:i: strains circulating in Europe. *PLoS One* 9:e89635. 10.1371/journal.pone.0089635 24586926 PMC3935914

[B34] Gaul S. B. Wedel S. Erdman M. M. Harris D. L. Harris I. T. Ferris K. E. (2007). Use of pulsed-field gel electrophoresis of conserved XbaI fragments for identification of swine *Salmonella* serotypes. *J. Clin. Microbiol.* 45 472–476. 10.1128/JCM.00962-06 17166969 PMC1829035

[B35] Gieraltowski L. Higa J. Peralta V. Green A. Schwensohn C. Rosen H. (2016). National outbreak of multidrug resistant *Salmonella* Heidelberg infections linked to a single poultry company. *PLoS One* 11:e0162369. 10.1371/journal.pone.0162369 27631492 PMC5025200

[B36] Hoffmann M. Zhao S. Pettengill J. Luo Y. Monday S. R. Abbott J. (2014). Comparative genomic analysis and virulence differences in closely related *Salmonella enterica* serotype Heidelberg isolates from humans, retail meats, and animals. *Genome Biol. Evol.* 6 1046–1068. 10.1093/gbe/evu079 24732280 PMC4040988

[B37] Iwamoto M. Reynolds J. Karp B. E. Tate H. Fedorka-Cray P. J. Plumblee J. R. (2017). Ceftriaxone-resistant nontyphoidal *Salmonella* from humans, retail meats, and food animals in the United States, 1996–2013. *Foodborne Pathog. Dis.* 14 74–83. 10.1089/fpd.2016.2180 27860517

[B38] Jones C. Stanley J. (1992). *Salmonella* plasmids of the pre-antibiotic era. *J. Gen. Microbiol.* 138 189–197. 10.1099/00221287-138-1-189 1556549

[B39] Kinross P. van Alphen L. Martinez Urtaza J. Struelens M. Takkinen J. Coulombier D. (2014). Multidisciplinary investigation of a multicountry outbreak of *Salmonella* stanley infections associated with Turkey meat in the European Union, August 2011 to January 2013. *Euro Surveill.* 19:20801. 10.2807/1560-7917.ES2014.19.19.20801 24852954

[B40] Li Q. Skyberg J. A. Fakhr M. K. Sherwood J. S. Nolan L. K. Logue C. M. (2006). Antimicrobial susceptibility and characterization of *Salmonella* isolates from processed bison carcasses. *Appl. Environ. Microbiol.* 72 3046–3049. 10.1128/AEM.72.4.3046-3049.2006 16598016 PMC1449034

[B41] Maka Ł. Maćkiw E. Ściezyńska H. Pawłowska K. Popowska M. (2014). Antimicrobial susceptibility of *Salmonella* strains isolated from retail meat products in Poland between 2008 and 2012. *Food Control* 36 199–204. 10.1016/j.foodcont.2013.08.025

[B42] Marasini D. Abo-Shama U. H. Fakhr M. K. (2016a). Complete genome sequences of *Salmonella enterica* serovars anatum and anatum var. 15 +, isolated from retail ground turkey. *Genome Announc.* 4:e01619-15. 10.1128/genomeA.01619-15 26798111 PMC4722278

[B43] Marasini D. Abo-Shama U. H. Fakhr M. K. (2016b). Whole-genome sequencing of *Salmonella enterica* subsp. *enterica* serovar Ouakam isolated from ground turkey. *Genome Announc.* 4:e01618-15. 10.1128/genomeA.01618-15 26798110 PMC4722277

[B44] Marasini D. Fakhr M. (2014). Exploring PFGE for detecting large plasmids in *Campylobacter jejuni* and *Campylobacter coli* isolated from various retail meats. *Pathogens* 3 833–844. 10.3390/pathogens3040833 25436507 PMC4282888

[B45] Mayer L. W. (1988). Use of plasmid profiles in epidemiologic surveillance of disease outbreaks and in tracing the transmission of antibiotic resistance. *Clin. Microbiol. Rev.* 1 228–243. 10.1128/CMR.1.2.228 2852997 PMC358044

[B46] Nair D. V. T. Johny A. K. (2017). Food grade Pimenta leaf essential oil reduces the attachment of *Salmonella enterica* Heidelberg (2011 Ground Turkey Outbreak Isolate) on to Turkey Skin. *Front. Microbiol.* 8:2328. 10.3389/fmicb.2017.02328 29234313 PMC5712355

[B47] Nair D. V. T. Thomas J. V. Noll S. Porter R. Johny A. K. (2018). Effect of various inoculum levels of multidrug-resistant *Salmonella enterica* serovar Heidelberg (2011 Ground Turkey Outbreak Isolate) on cecal colonization, dissemination to internal organs, and deposition in skeletal muscles of commercial Turkeys after e. *Front. Microbiol.* 8:2680 10.3389/fmicb.2017.02680 PMC5771268 29379476

[B48] Nde C. W. Fakhr M. K. Doetkott C. Logue C. M. (2008). An evaluation of conventional culture, invA PCR, and the real-time PCR iQ-check kit as detection tools for *Salmonella* in naturally contaminated premarket and retail turkey. *J. Food Prot.* 71 386–391. 10.4315/0362-028X-71.2.386 18326192

[B49] Nisar M. Kassem I. I. Rajashekara G. Goyal S. M. Lauer D. Voss S. (2017). Genotypic relatedness and antimicrobial resistance of *Salmonella* Heidelberg isolated from chickens and turkeys in the midwestern United States. *J. Vet. Diagn. Investig.* 29 370–375. 10.1177/1040638717690784 28430086

[B50] Noormohamed A. Fakhr M. (2013). A higher prevalence rate of campylobacter in retail beef livers compared to other beef and pork meat cuts. *Int. J. Environ. Res. Public Health* 10 2058–2068. 10.3390/ijerph10052058 23698698 PMC3709364

[B51] Noormohamed A. Fakhr M. K. (2012). Incidence and antimicrobial resistance profiling of campylobacter in retail chicken livers and gizzards. *Foodborne Pathog. Dis.* 9 617–624. 10.1089/fpd.2011.1074 22545960

[B52] Noormohamed A. Fakhr M. K. (2014). Prevalence and antimicrobial susceptibility of Campylobacter spp. in Oklahoma conventional and organic retail poultry. *Open Microbiol. J.* 8 130–137. 10.2174/1874285801408010130 25408778 PMC4235082

[B53] Paudyal N. Pan H. Li X. Fang W. Yue M. (2018). Antibiotic resistance in *Salmonella* enteritidis isolates recovered from chicken, chicken breast, and humans through national antimicrobial resistance monitoring system between 1996 and 2014. *Foodborne Pathog. Dis.* 10.1089/fpd.2017.2402 [Epub ahead of print]. 29927626

[B54] Rahn K. De Grandis S. A. Clarke R. C. McEwen S. A. Galán J. E. Ginocchio C. (1992). Amplification of an invA gene sequence of *Salmonella* typhimurium by polymerase chain reaction as a specific method of detection of *Salmonella*. *Mol. Cell. Probes* 6 271–279. 10.1016/0890-8508(92)90002-F 1528198

[B55] Rotgers R. Casadesüs J. (1999). The virulence plasmids of *Salmonella*. *Int. Microbiol.* 2 177–184. 10.2436/im.v2i3.9210 10943411

[B56] Rychlik I. Gregorova D. Hradecka H. (2006). Distribution and function of plasmids in *Salmonella enterica*. *Vet. Microbiol.* 112 1–10. 10.1016/j.vetmic.2005.10.030 16303262

[B57] Sajid S. U. Schwarz S. (2009). Plasmid fingerprinting and virulence gene detection among indigenous strains of *Salmonella enterica* serovar Enteritidis. *J. Ayub Med. Coll.* 21 83–86. 20524477

[B58] Sallam K. I. Mohammed M. A. Hassan M. A. Tamura T. (2014). Prevalence, molecular identification and antimicrobial resistance profile of *Salmonella* serovars isolated from retail beef products in Mansoura, Egypt. *Food Control* 38 209–214. 10.1016/j.foodcont.2013.10.027

[B59] Sanad Y. M. Johnson K. Park S. H. Han J. Deck J. Foley S. L. (2016). Molecular characterization of *Salmonella enterica* serovars isolated from a turkey production facility in the absence of selective antimicrobial pressure. *Foodborne Pathog. Dis.* 13 80–87. 10.1089/fpd.2015.2002 26653998

[B60] Scallan E. Hoekstra R. M. Angulo F. J. Tauxe R. V. Widdowson M. A. Roy S. L. (2011). Foodborne illness acquired in the United States-Major pathogens. *Emerg. Infect. Dis.* 17 7–15. 10.3201/eid1701.P11101 21192848 PMC3375761

[B61] Sharma C. S. Dhakal J. Nannapaneni R. (2015). Efficacy of lytic bacteriophage preparation in reducing *Salmonella* in vitro, on turkey breast cutlets, and on ground turkey. *J. Food Prot.* 78 1357–1362. 10.4315/0362-028X.JFP-14-585 26197288

[B62] Sjölund-Karlsson M. Howie R. Rickert R. Krueger A. Tran T. T. Zhao S. (2010). Plasmid-mediated quinolone resistance among non-typhi *Salmonella enterica* isolates, USA. *Emerg. Infect. Dis.* 16 1789–1791. 10.3201/eid1611.100464 21029547 PMC3294515

[B63] Soufi L. Sáenz Y. de Toro M. Salah Abbassi M. Rojo-Bezares B. Vinué L. (2012). Phenotypic and genotypic characterization of *Salmonella enterica* recovered from poultry meat in Tunisia and identification of new genetic traits. *Vector Borne Zoonotic Dis.* 12 10–16. 10.1089/vbz.2011.0667 21919733 PMC3249895

[B64] Tafida S. Y. Kabir J. Kwaga J. K. P. Bello M. Umoh V. J. Yakubu S. E. (2013). Occurrence of *Salmonella* in retail beef and related meat products in Zaria, Nigeria. *Food Control* 32 119–124. 10.1016/j.foodcont.2012.11.005

[B65] Tenover F. C. Arbeit R. D. Goering R. V. Mickelsen P. A. Murray B. E. Persing D. H. (1995). Interpreting chromosomal DNA restriction patterns produced by pulsed-field gel electrophoresis: criteria for bacterial strain typing. *J. Clin. Microbiol.* 33 2233–2239. 7494007 10.1128/jcm.33.9.2233-2239.1995 PMC228385

[B66] Thai T. H. Hirai T. Lan N. T. Yamaguchi R. (2012). Antibiotic resistance profiles of *Salmonella* serovars isolated from retail pork and chicken meat in North Vietnam. *Int. J. Food Microbiol.* 156 147–151. 10.1016/j.ijfoodmicro.2012.03.016 22497836

[B67] Van T. T. H. Nguyen H. N. K. Smooker P. M. Coloe P. J. (2012). The antibiotic resistance characteristics of non-typhoidal *Salmonella enterica* isolated from food-producing animals, retail meat and humans in South East Asia. *Int. J. Food Microbiol.* 154 98–106. 10.1016/j.ijfoodmicro.2011.12.032 22265849

[B68] White D. G. Zhao S. Sudler R. Ayers S. Friedman S. Chen S. (2001). The isolation of antibiotic-resistant *Salmonella* from retail ground meats. *N. Engl. J. Med.* 345 1147–1154. 10.1056/NEJMoa010315 11642230

[B69] Wittum T. E. (2012). The challenge of regulating agricultural ceftiofur use to slow the emergence of resistance to extended-spectrum cephalosporins. *Appl. Environ. Microbiol.* 78 7819–7821. 10.1128/AEM.01967-12 22961892 PMC3485946

[B70] Yang B. Qu D. Zhang X. Shen J. Cui S. Shi Y. (2010). Prevalence and characterization of *Salmonella* serovars in retail meats of marketplace in Shaanxi, China. *Int. J. Food Microbiol.* 141 63–72. 10.1016/j.ijfoodmicro.2010.04.015 20493570

[B71] Yang B. Wang Q. Cui S. Wang Y. Shi C. Xia X. (2014). Characterization of extended-spectrum beta-lactamases-producing *Salmonella* strains isolated from retail foods in Shaanxi and Henan Province, China. *Food Microbiol.* 42 14–18. 10.1016/j.fm.2014.02.003 24929711

[B72] Yildirim Y. Gonulalan Z. Pamuk S. Ertas N. (2011). Incidence and antibiotic resistance of *Salmonella* spp. on raw chicken carcasses. *Food Res. Int.* 44 725–728. 10.1016/j.foodres.2010.12.040

[B73] Zhao S. Dermott P. F. M. C. Friedman S. Abbott J. Ayers S. Glenn A. (2006). Antimicrobial resistance and genetic relatedness among *Salmonella* from retail foods of animal origin: NARMS retail meat surveillance. *Foodborne Pathog. Dis.* 3 106–117. 10.1089/fpd.2006.3.106 16602986

[B74] Zhao S. White D. G. Friedman S. L. Glenn A. Blickenstaff K. Ayers S. L. (2008). Antimicrobial resistance in *Salmonella enterica* serovar Heidelberg isolates from retail meats, including poultry, from 2002 to 2006. *Appl. Environ. Microbiol.* 74 6656–6662. 10.1128/AEM.01249-08 18757574 PMC2576681

[B75] Zou W. Chen H. C. Hise K. B. Tang H. Foley S. L. Meehan J. (2013). Meta-analysis of pulsed-field gel electrophoresis fingerprints based on a constructed *Salmonella* database. *PLoS One* 8:e59224. 10.1371/journal.pone.0059224 23516614 PMC3597626

[B76] Zou W. Lin W. J. Foley S. L. Chen C. H. Nayak R. Chen J. J. (2010). Evaluation of pulsed-field gel electrophoresis profiles for identification of *Salmonella* serotypes. *J. Clin. Microbiol.* 48 3122–3126. 10.1128/JCM.00645-10 20631109 PMC2937721

